# PM_2.5_-Induced Cardiac Structural Modifications and Declined Pro-Survival Signalling Pathways Are Responsible for the Inefficiency of GSK-3β Inhibitor in Attenuating Myocardial Ischemia-Reperfusion Injury in Rats

**DOI:** 10.3390/cells12162064

**Published:** 2023-08-15

**Authors:** Bhavana Sivakumar, Nemat Ali, Sheikh F. Ahmad, Ahmed Nadeem, Mohammad Waseem, Gino A. Kurian

**Affiliations:** 1 Vascular Biology Laboratory, School of Chemical and Biotechnology, SASTRA Deemed University, Thanjavur 613401, Tamil Nadu, India; bhavana@scbt.sastra.edu; 2 Department of Pharmacology and Toxicology, College of Pharmacy, King Saud University, Riyadh 11451, Saudi Arabia; nali1@ksu.edu.sa (N.A.); fashaikh@ksu.edu.sa (S.F.A.); anadeem@ksu.edu.sa (A.N.); 3 Department of Pharmaceutical Sciences, School of Pharmacy, University of Maryland Eastern Shore, Princess Anne, MD 21853, USA; mwaseem@umes.edu

**Keywords:** PM_2.5_, cardiovascular diseases, diesel particulate matter, GSK 3β, SB216763, ischemia-reperfusion injury, mitochondria, biomarker

## Abstract

Circulatory GSK3β is recognized as a biomarker and therapeutic target for diseases, including myocardial diseases. However, its potential as a target for myocardial ischemia-reperfusion injury (IR) in the presence of PM_2.5_ exposure is unclear. Wistar rats underwent IR following either a 21-day or single exposure to PM_2.5_ at a concentration of 250 µg/m^3^. The effects of GSK3β inhibitor on cardiac physiology, tissue injury, mitochondrial function, and the PI3K/AKT/GSK3β signalling axis were examined. The inhibitor was not effective in improving hemodynamics or reducing IR-induced infarction in the myocardium exposed to PM_2.5_ for 21 days. However, for a single-day exposure, the inhibitor showed potential in mitigating cardiac injury. In normal hearts undergoing IR, the inhibitor activated the PI3K/AKT signalling pathway, improved mitochondrial function, and reduced oxidative stress. These positive effects were not observed in PM_2.5_-exposed rats. Furthermore, the inhibitor stimulated autophagy in hearts exposed to PM_2.5_ for 21 days and subjected to IR, resulting in increased mTOR expression and decreased AMPK expression. In normal hearts and those exposed to a single dose of PM_2.5_, the inhibitor effectively activated the PI3K/Akt/AMPK axis. These findings suggest that GSK3β may not be a reliable therapeutic target for IR in the presence of chronic PM_2.5_ exposure.

## 1. Introduction

Air pollution is a major global health concern, especially due to exposure to fine particulate matter (PM_2.5_) [1]. This exposure has been linked to cardiovascular diseases, as PM_2.5_ is a complex mixture of toxic constituents that can negatively affect cardiac function and promote cardiac infarcts [2]. Numerous epidemiological studies have consistently shown a positive association between PM_2.5_ exposure and increased cardiovascular morbidity and mortality [3,4,5]. While the relationship between PM_2.5_ exposure and cardiovascular disease is well established, the exact mechanisms by which PM_2.5_ exerts its harmful effects on the cardiovascular system are not fully understood. PM_2.5_ consists of various toxic constituents, such as heavy metals, organic compounds, and combustion by-products [6], which can induce oxidative stress, inflammation, and endothelial dysfunction, contributing to the development and progression of cardiovascular diseases [7]. Additionally, PM_2.5_ has been shown to impair cardiac physiological function and promote cardiac infarcts [8]. However, further investigation is required to understand the specific molecular and cellular pathways involved in PM_2.5_-induced cardiotoxicity.

One potential mechanism through which PM_2.5_ may exert its detrimental effects on the cardiovascular system is by disrupting signalling pathways associated with cardiac protection and repair. The glycogen synthase kinase-3 beta (GSK3β) signalling pathway plays a critical role in cell survival, apoptosis, and inflammation [9,10,11]. Activation of GSK3β has been associated with apoptosis and inflammatory responses in various cell types, while its inhibition has been linked to cardio-protection [12,13,14]. However, the involvement of the GSK3β signalling pathway in PM_2.5_-induced cardiotoxicity requires further investigation.

Furthermore, the interaction between PM_2.5_ exposure and myocardial ischemia-reperfusion (IR) injury, a condition characterized by the temporary interruption and subsequent restoration of blood flow to the heart, presents an additional challenge [15,16]. IR injury is a significant contributor to myocardial damage, and understanding its pathophysiology is crucial to improving clinical outcomes [17]. PM_2.5_ exposure, known to induce systemic inflammation and oxidative stress, has the potential to exacerbate myocardial injury following IR [15,16]. However, the underlying molecular mechanisms involved in the interplay between PM_2.5_ exposure, IR injury, and the GSK3β signalling axis, as well as their impact on cardiac function and mitochondrial dysfunction, remain unclear.

Although several studies have investigated the cardiovascular effects of PM_2.5_ exposure, there is a lack of research specifically focusing on the role of GSK3β signalling and its modulation in PM_2.5_-induced cardiotoxicity. Additionally, the interactions between PM_2.5_ exposure and IR injury, particularly in relation to cardiac function and mitochondrial dysfunction, have not been extensively explored. Therefore, this study aimed to use an animal model to examine the effects of SRM 2975, a source of PM_2.5_, on Wistar rats. The rats were exposed to PM_2.5_ followed by the administration of GSK3β inhibitor (SB216763) and subsequent subjection to IR injury. The evaluations included cardiac physiological functions, injury markers, inflammation, apoptosis, mitochondrial function, and the expression of key molecules, i.e., the PI3K/AKT/mTOR/AMPK/GSK3β signalling axis. Furthermore, the effectiveness of SB216763 in attenuating PM2.5-induced cardiotoxicity and mitigating the impact of IR injury was assessed.

In summary, this study aimed to elucidate the molecular mechanisms underlying PM_2.5_-induced cardiotoxicity, with a specific focus on the involvement of the GSK3β signalling pathway and mitochondrial dysfunction. By investigating the interplay between PM_2.5_ exposure and IR injury, and their impact on cardiac function, this research contributes to our understanding of air-pollution-related cardiovascular diseases and guides the development of targeted interventions to mitigate their detrimental effects.

## 2. Materials and Methods

### 2.1. Chemicals and Reagents

SRM-2975 was procured from the National Institute of Standards and Technology, Gaithersburg, MD, USA. All chemicals and reagents were purchased from Sigma-Aldrich (St. Louis, MO, USA) unless specifically described. Antibodies were purchased from Cell signalling technology, Danvers, Massachusetts, USA.

### 2.2. Animals

Animal experiments at SASTRA Deemed University, Thanjavur, India, were carried out in compliance with the regulations set by the Committee for the Purpose of Control and Supervision of Experiments on Animals (CPCSEA) and approved by the Institutional Animal Ethical Committee (IAEC) (752/SASTRA/IAEC/RPP). Female Wistar rats, weighing between 200–250 g, were kept in a controlled environment with regulated temperature and humidity (23 ± 2 °C, 60–70%, 12 h light/dark cycle), and provided with unrestricted access to food and water.

### 2.3. IR Induction and Hemodynamics Assessment

To induce IR injury, the hearts were subjected to 30 min of ischemia by halting the flow of KH buffer, followed by 60 min of reperfusion by resuming the buffer perfusion. Throughout the entire experiment, hemodynamic parameters were continuously monitored using AD instruments’ Lab chart software (LAB CHART PRO) from Australia. At the end of the experiment, the hearts were collected and preserved for subsequent analysis [16].

### 2.4. Experimental Protocol

The animal exposure to PM_2.5_ was conducted using a modified whole-body animal exposure model, as detailed in our previous publication [15]. The temperature, pressure, and flow rate within the chamber were maintained at optimal levels. To monitor the concentrations of PM, oxygen (O_2_), and carbon monoxide (CO), as well as the temperature and humidity inside the chamber, a PRANA air-sourced CAIR air quality monitor was employed. The experimental animals used in the present study were divided into two major groups: Group A (composed of 42 rats), exposed to PM_2.5_ for 21 days; and Group B (composed of 42 rats), exposed to PM_2.5_ for 1 day. Group A and B were further subdivided into 7 groups composed of 6 animals per group.

**Group A:** (21-day exposure study): 1: Normal; 2: IR control; 3: PM_2.5_ exposure control (PM_C): Exposure to PM_2.5_ at a concentration of 250 µg/m^3^ for 3 h daily for 21 days; 4: PM_2.5_ exposure followed by IR induction (PM_IR): Exposure to PM_2.5_ followed by IR induction; 5: GSK3β_IR: 0.7 mg/kg of GSK3β inhibitor: SB216763 was administered intraperitoneally followed by IR induction; 6: PM+ GSK3β_C: Exposure to PM_2.5_ at a concentration of 250 µg/m^3^ for 3 h daily for 21 days followed by 0.7 mg/kg of SB216763 administration and normal perfusion for 120 min; 7: PM+ GSK3β_IR: Exposure to PM_2.5_ at a concentration of 250 µg/m^3^ for 3 h daily for 21 days followed by SB216763 administration and IR induction.

**Group B:** (1-day exposure study): Normal; 2: IR control; 3: PM_2.5_ exposure control (PM 1day_C): Exposure to PM_2.5_ at a concentration of 250 µg/m^3^ for 1 h; 4: PM_2.5_ exposure followed by IR induction (PM 1day_IR): Exposure to PM_2.5_ followed by IR induction; 5: GSK3β_IR: 0.7 mg/kg of GSK3β inhibitor: SB216763 was administered intraperitoneally followed by IR induction; 6: PM 1day+ GSK3β_C: Exposure to PM_2.5_ at a concentration of 250 µg/m^3^ for 1 h followed by 0.7 mg/kg of SB216763 administration and normal perfusion for 120 min; 7: PM 1 day + GSK3β_IR: Exposure to PM_2.5_ at a concentration of 250 µg/m^3^ for 1 h followed by SB216763 administration and IR induction.

### 2.5. Cardiac Injury Assessment

To evaluate cardiac injury, the levels of lactate dehydrogenase (LDH) and creatine kinase (CK) were measured in tissue homogenate. Additionally, as described before, LDH, CK, and caspase-3 activity were assessed. Structural assessment of the injury involved staining heart sections with triphenyl tetrazolium chloride (TTC), and the percentage of the infarcted area was calculated using the ImageJ software (1.53k) (NIH-USA) [18].

### 2.6. Western Blot

To ensure optimal sample processing, all samples were handled at 4 °C. In brief, myocardial tissue samples were homogenized using an ice-cold RIPA lysis buffer, and the protein concentration was determined using Lowry’s method. Equal concentrations of proteins were then prepared in SDS lysis buffer, followed by denaturation for 15 min at 80 °C. Subsequently, the proteins were resolved in a 5% stacking gel and a 10% resolving gel for SDS-polyacrylamide gel electrophoresis (SDS-PAGE). The resolved proteins were transferred onto PVDF membranes with a pore size of 0.45 μm. The membranes were blocked using 5% BSA in TBST for 1 h. After repeated washes with TBST for 15 min, the membranes were probed overnight at 4 °C with primary antibodies (Dilution 1:2000), including p-PI3K (Tyr 458/Tyr 199) (CST #4228), PI3K (CST #4225), p-AKT (Ser 473) (CST #4060), Total Akt (CST #C67E7), mTOR (CST #2972), p-mTOR (CST #5536), AMPK (#2532), p-AMPK (#2535), cleaved caspase-3 (#9661), 7 (#9491), 9 (#9505), GSK3B (#9315), pGSk3B (#9336), ATG 3 (#3415), 5 (#12994), 7 (#8558), 12 (#4180), Beclin 1 (#3738), LC3A/B (#4108), and Beta-actin (CST #13E5). Following incubation, the membranes were washed three times with TBST for 15 min and then incubated with secondary antibodies (Dilution: 1:3000), such as anti-rabbit secondary antibody (CST #7074) in TBST for 1 h at room temperature. After another three washes with TBST, the blot membranes were imaged using a chemiluminescent detection system (ECL, BioRad, CA, USA) in a Chemi-Doc XRS (BioRad, USA). The relative expression of the bands was quantified using Quantity-One 4.6 version, image analysis software (BioRad, CA, USA) [19].

### 2.7. Mitochondrial Isolation, Bioenergetics, and Oxidative Stress Assessment

Isolation of mitochondria was carried out using the differential centrifugal technique as previously reported [20]. The activity of mitochondrial electron transport chain (ETC) enzymes, including NADH-oxidoreductase (NQR), succinate decylubiquinone DCPIP reductase (SQR), ubiquinol cytochrome c reductase (QCR), and cytochrome c oxidase (COX), was assessed using a Synergy H1 multimode reader (BioTek, Vermont, USA). To measure complex I activity, decylubiquinone was utilized, while complex II activity was determined by measuring DCIP reduction at 600 nm. The activity of complex III (cytochrome c reductase) was measured by monitoring the rate of cytochrome c reduction at 550 nm, and complex IV activity (cytochrome c oxidase) was evaluated by observing the rate of cytochrome c oxidation at 550 nm. GSH/GSSG levels were measured as previously described [21].

### 2.8. ELISA Assay

The levels of TNF-a and IL-6 in the tissue homogenate were estimated using ELISA kits purchased from Krishgen Biosystems, India.

### 2.9. Statistical Analysis

The data are presented as mean ± standard deviation (SD). Post hoc analysis consisting of one-way ANOVA, followed by Dunnett’s test, was used for comparison among different groups using GraphPad Prism 8 software (GraphPad Software, LaJolla, CA, USA). Statistical significance was represented as *^$^ *p* < 0.05.

## 3. Results

### 3.1. GSK3β Inhibitor Effectively Attenuated IR-Induced Cardiac Injury in Rat Hearts Exposed to PM_2.5_ for Shorter Duration

**Figure 1:**
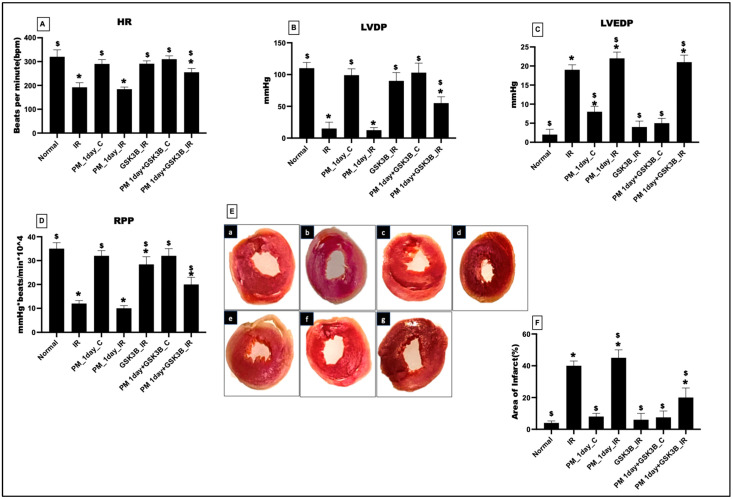
Cardiac hemodynamic parameters measured via Lab chart software. (**A**) Heart rate (HR); (**B**) Left ventricular developed pressure (LVDP); (**C**) Left ventricular end-diastolic pressure (LVEDP); (**D**) rate pressure product (RPP); (**E**) Representative light microscopy photograph of TTC staining of different groups (a: Normal; b: IR; c: PM_1day_C; d: PM_1day_IR; e: GSK3β_IR; f: PM1day+ GSK3β_C; g: PM1day+ GSK3β_IR); (**F**) Infarct size measurement via ImageJ software; * *p* < 0.05 vs. normal, ^$^ *p* < 0.05 vs. IR control. Groups include: Normal; IR; PM_1day_C (PM_2.5_ exposure for a single day followed by normal perfusion); PM_1day_IR (PM_2.5_ exposure for a single day followed by IR induction); GSK3β_IR (SB216763 administration followed by IR induction); PM1day+ GSK3β_C (PM_2.5_ exposure followed by SB216763 administration and normal perfusion); PM1day+ GSK3β_IR (PM_2.5_ exposure followed by SB216763 administration and IR induction), (n = 6 per group).

Figure 1 displays the impact of a single exposure to PM_2.5_ on the myocardium. The cardiac hemodynamic parameters showed no significant changes from the normal and IR control. The HR, LVDP, and RPP showed an insignificant change in the PM_1day_C (vs. normal control). Even with IR induction, there was no significant change in these indices from the IR control. However, administration of SB216763 improved the HR (25%), LVDP (72%), LVEDP (10%), and RPP (40%) in the PM1_day+GSK3B_IR from the IR control. In support to these findings, infarct size measurement using TTC displayed no significant cardiac injury in the PM_1day_C and PM_1 day_IR from the normal control and IR control groups. However, SB216763 administration reduced the cardiac infarct area (50%) in PM_1 day_IR.

### 3.2. Long-Term Exposure to PM_2.5_ Abrogated the Potential of GSK3β Inhibitor to Ameliorate IR Injury in Rat Hearts

**Figure 2:**
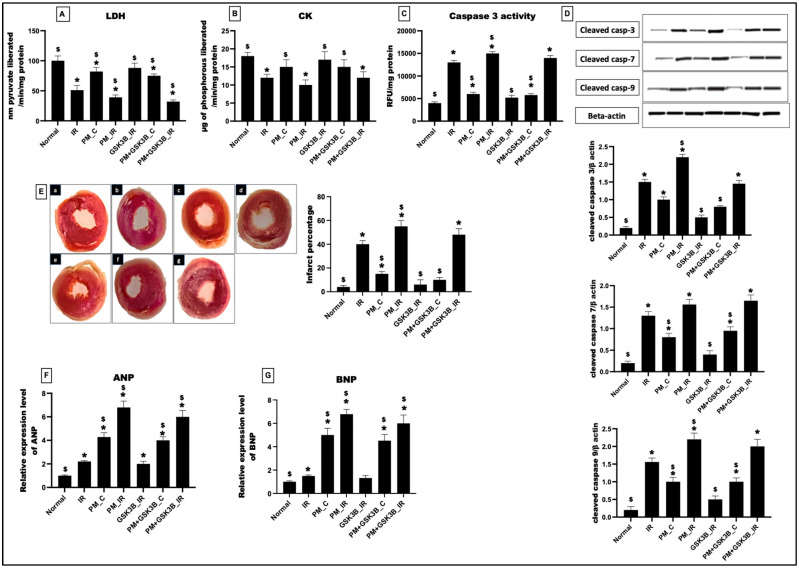
Cardiac injury assessment: (**A**) LDH level in tissue; (**B**) CK level in tissue; (**C**) Caspase-3 activity; (**D**) Protein expression of cleaved caspase-3, 7, 9 and quantification using ImageJ software; (**E**) Representative light microscopy photograph of TTC staining of different groups and Infarct size measurement via ImageJ software (a: Normal; b: IR; c: PM_C; d: PM_IR; e: GSK3β_IR; f: PM+ GSK3β_C; g: PM+ GSK3β_IR. (**F**) ANP expression in the myocardium; (**G**) BNP expression in the myocardium, * *p* < 0.05 vs. normal, ^$^ *p* < 0.05 vs. IR control. Groups include: Normal; IR; PM_C (PM_2.5_ exposure for 21 days followed by normal perfusion); PM_IR (PM_2.5_ exposure for 21 days followed by IR induction); GSK3β_IR (SB216763 administration followed by IR induction); PM+ GSK3β_C (PM_2.5_ exposure followed by SB216763 administration and normal perfusion); PM+ GSK3β_IR (PM_2.5_ exposure followed by SB216763 administration and IR induction), (n = 6 per group).

As observed in Figure 2A,B, exposure to PM_2.5_ for 21 days resulted in an 18% and 17% decline in the LDH and CK levels, respectively, in the myocardia of the PM_C group. This was supported by and increased infarct percentage of 73% (vs. normal control). Caspase-3 activity revealed an increased apoptotic injury percentage of 33% from the normal control. In support of this, the caspase-3, 7, 9 levels also showed a significant upregulation of 80%, 75% and 80%, respectively. Further analysis of the hypertrophy markers: ANP and BNP revealed significant increases of 76% and 80%, respectively, from the normal control. However, in the presence of SB216763, the myocardial infraction and the levels of cardiac injury markers displayed no significant change from the PM_C group. The caspase activity and protein expression also showed no significant changes from the normal control. Administration of SB216763 failed to attenuate cardiac hypertrophy in the PM+GSK3B_C group.

Similarly, in the presence of IR, SB216763 administration resulted in no significant changes in the LDH (17%), CK (19%), and caspase-3 activity (6%) in the PM+GSK3B_IR group (vs. PM_IR). Infarct size measurement via TTC revealed a non-significant decline in the cardiac infarct size by 12% in PM+GSK3B_IR from the PM_IR control. This was supported by the ANP and BNP expression, where administration of GSK3B inhibitor failed to reduce hypertrophy in the PM+GSK3B_C and PM+GSK3B_IR groups.

### 3.3. Long-Term Exposure to PM_2.5_ Abrogated the Potential of GSK3β Inhibitor to Ameliorate Cardiac Hemodynamics in Rat Hearts

**Figure 3:**
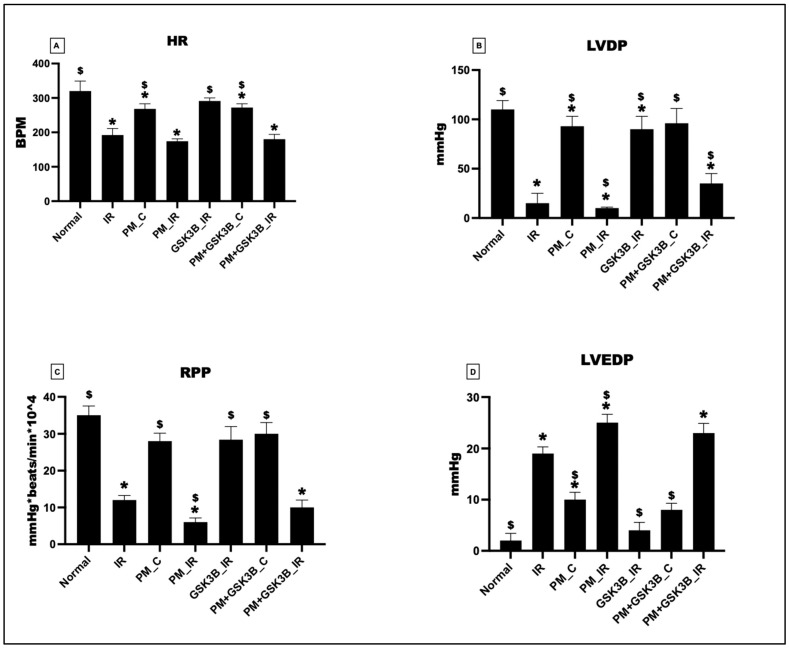
Cardiac hemodynamic parameters measured via Lab chart software. (**A**) Heart rate (HR); (**B**) Left ventricular end-diastolic pressure (LVEDP); (**C**) rate pressure product (RPP); (**D**) Left ventricular developed pressure (LVDP), * *p* < 0.05 vs. normal, ^$^ *p* < 0.05 vs. IR control. Groups include: Normal; IR; PM_C (PM_2.5_ for 21 days exposure followed by normal perfusion); PM_IR (PM_2.5_ exposure followed for 21 days by IR induction); GSK3β_IR (SB216763 administration followed by IR induction); PM+ GSK3β_C (PM_2.5_ exposure followed by SB216763 administration and normal perfusion); PM+ GSK3β_IR (PM_2.5_ exposure followed by SB216763 administration and IR induction), (n = 6 per group).

The functioning of the cardiac system is assessed through the evaluation of cardiac hemodynamics. As depicted in Figure 3, exposure to PM_2.5_ for 21 days resulted in a deterioration of cardiac hemodynamic indices such as heart rate (HR), left ventricular developed pressure (LVDP), left ventricular end-diastolic pressure (LVEDP), and rate-pressure product (RPP). In the PM_C group, HR, LVDP, and RPP decreased by 16%, 15%, and 20%, respectively, and LVEDP increased by 80%. The addition of SB216763 insignificantly attenuated cardiac hemodynamics in the PM+SB216763_C group (HR: 2%, LVDP: 3%, RPP: 6%, LVEDP: 2%). Subsequently, IR induction further decreased the hemodynamic indices in the PM_IR group. HR, LVDP, and RPP reduced by 10%, 50%, and 33%, respectively, from the IR control. However, the administration of SB216763 failed to improve the hemodynamics in PM_2.5_-exposed hearts subjected to IR.

### 3.4. Long-Term Exposure to PM_2.5_ Inactivated the PI3K/AKT/mTOR/GSK3B/AMPK Signalling Axis in IR Rat Hearts Even in the Presence of GSK3β Inhibitor

**Figure 4:**
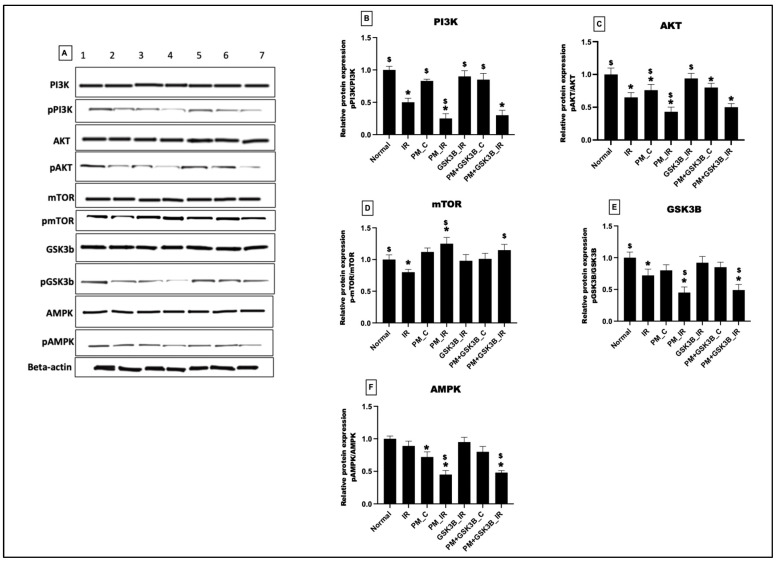
(**A**) Western blot analysis. Quantification of (**B**) PI3K, (**C**) AKT, (**D**) mTOR, (**E**) GSK3β, (**F**) AMPK using ImageJ software, * *p* < 0.05 vs. normal, ^$^ *p* < 0.05 vs. IR control. Groups include: 1: Normal; 2: IR, 3: PM_C (PM_2.5_ exposure for 21 days followed by normal perfusion); 4: PM_IR (PM_2.5_ exposure for 21 days followed by IR induction); 5: GSK3β_IR (SB216763 administration followed by IR induction); 6: PM+ GSK3β_C (PM_2.5_ exposure followed by SB216763 administration and normal perfusion); 7: PM+ GSK3β_IR (PM_2.5_ exposure followed by SB216763 administration and IR induction).

Figure 4 illustrates the impact of PM_2.5_ exposure on cardiac pro-survival signalling pathways. In the PM_C group, PM_2.5_ exposure significantly downregulated the PI3K, AKT, and AMPK pathways. Following IR induction, the PM_IR group displayed a significant downregulation of PI3K, AKT, GSK3β, and AMPK signalling pathways compared with the IR control group. Administration of GSK3B inhibitor showed attenuation of upstream pathways, such as PI3K and AKT, along with AMPK expression in the GSK3β_IR group. However, the inhibitor did not improve the pro-survival signalling axis in the PM+ GSK3β_C and PM+ GSK3β_IR groups. Conversely, the mTOR pathway was upregulated in the PM_C and PM_IR groups, indicating increased hypertrophy. GSK3B inhibitor did not act as a therapeutic target to prevent hypertrophy in the PM+ GSK3β_C and PM+ GSK3β_IR groups.

### 3.5. Long-Term Exposure to PM_2.5_ Activated the mTOR Dependent Autophagy in IR Rat Hearts Even in the Presence of GSK3β Inhibitor

**Figure 5:**
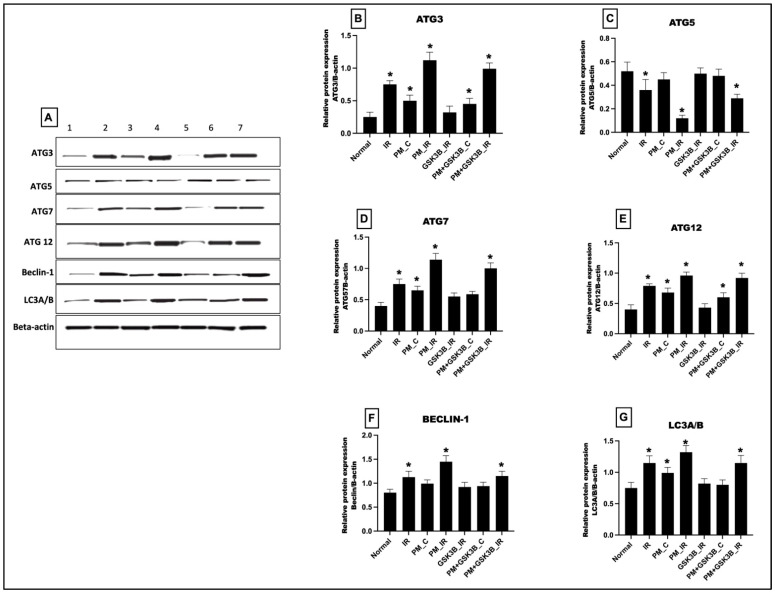
(**A**) Western blot analysis. Quantification of (**B**) ATG3, (**C**) ATG5, (**D**) ATG7, (**E**) ATG12, (**F**) Beclin-1, (**G**) LC3A/B using ImageJ software, * *p* < 0.05 vs. normal. Groups include: 1: Normal; 2: IR, 3: PM_C (PM_2.5_ exposure for 21 days followed by normal perfusion); 4: PM_IR (PM_2.5_ exposure for 21 days followed by IR induction); 5: GSK3β_IR (SB216763 administration followed by IR induction); 6: PM+ GSK3β_C (PM_2.5_ exposure followed by SB216763 administration and normal perfusion); 7: PM+ GSK3β_IR (PM_2.5_ exposure followed by SB216763 administration and IR induction).

As observed in Figure 5, 21 days of PM_2.5_ exposure significantly increased the cardiac autophagy. Autophagy markers like ATG 3, 7, 12, beclin-1, and LC3A/B showed a significant upregulation in the PM_C group. After IR induction, the autophagy expression was further increased significantly from the normal control. SB216763 could reduce autophagy in IR rat hearts but not in PM_2.5_-exposed myocardia.

### 3.6. Long-Term Exposure to PM_2.5_ Induced Oxidative Stress, Mitochondrial Dysfunction and Inflammation in IR Rat Hearts Even in the Presence of GSK3β Inhibitor

**Figure 6:**
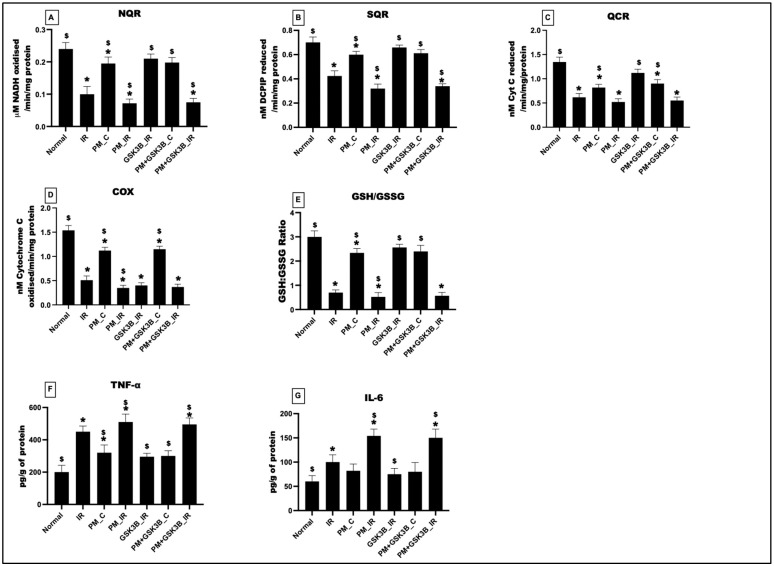
(**A**) NQR, (**B**) SQR, (**C**) QCR, (**D**) COX, (**E**) GSH/GSSG ratio determination in cardiac mitochondria of different groups. Determination of tissue activity of (**F**) TNF-a, (**G**) IL-6. * *p* < 0.05 vs. normal, ^$^ *p* < 0.05 vs. IR control. Groups include: Normal; IR; PM_C (PM_2.5_ exposure for 21 days followed by normal perfusion); PM_IR (PM_2.5_ exposure for 21 days followed by IR induction); GSK3β_IR (SB216763 administration followed by IR induction); PM+ GSK3β_C (PM_2.5_ exposure followed by SB216763 administration and normal perfusion); PM+ GSK3β_IR (PM_2.5_ exposure followed by SB216763 administration and IR induction).

Figure 6 displays the impact of PM_2.5_ exposure on mitochondrial bioenergetics, oxidative stress, and inflammation. In support of the above findings, PM_2.5_ exposure for 21 days decreased the activity of NQR, SQR, QCR, and COX in the PM_C group (vs. normal control). IR induction further deteriorated the activity of the complexes to 28%, 24%, 14%, and 30%, respectively, from the IR control. SB216763 administration could not activate the complexes and there was no significant change in the mitochondrial bioenergetics function in the PM+GSK3B_C and PM+GSK3B_IR groups. The GSH/GSSG levels showed a significant decline in the PM_C and PM_IR groups, and SB216763 administration led to an insignificant increase in the ratio by 18% in the PM+GSk3B_IR group (vs. IR control). Analysis of inflammatory markers TNF-a and IL-6 revealed a significant increase in their levels in the PM_C and PM_IR groups and, as explained above, SB216763 was not a potential inhibitor of inflammatory markers.

## 4. Discussion

The therapeutic potential of GSK3β inhibitor in the management of cardiovascular diseases is well known, where this molecule modulates the activity of pro-survival signalling pathways and preserves mitochondrial function [22]. In the present study, we demonstrated that the protective effect of the GSK3β inhibitor SB216763 failed to attenuate IR injury in rat hearts when the animals were pre-exposed to PM_2.5_ for 21 days. Furthermore, we showed that the protective effect of SB216763 was intact if the animals were exposed to a single dose of PM_2.5_. We found structural modifications in the rat hearts with 21 days of PM_2.5_ exposure that resulted in cardiac hypertrophy, which was absent in hearts with a single day of exposure. To uncover the molecular mechanism, we explored the activity of pro-survival signalling pathways. Unlike the SB216763-administered IR rat hearts, in PM_2.5_-exposed animals administered with the inhibitor, the PI3K/AKT/AMPK signalling pathways failed to undergo recovery. Similarly, mTOR-linked autophagy was high in PM_2.5_-exposed rat hearts subjected to IR challenge which could not be downregulated even with the administration of SB216763. Additionally, GSK3β inhibition could not effectively reduce the IR mediators like ROS and mitochondrial dysfunction in rats that underwent long-term exposure. GSK3β signalling is involved in various pathological conditions in the heart, namely, ischemic injury, fibrosis, cardiomyocyte proliferation and ageing [14]. GSK3β is involved in different signalling cascades in the heart that include regulation of NF-AT autophagy and metabolism, regulation of the D and E types of cyclin, and regulation of the proliferation and metabolism mediated by myc [23]. Similarly, GSK3β can negatively regulate profibrotic TGFß1 signalling after binding to SMAD-3 [24]. Moreover, GSK3β can regulate canonical Wnt signalling as well [25]. In the present study, we used SB216763 as the GSK3β inhibitor that can block both the α and ß forms of GSK3β.

The depletion of ATP and the accumulation of lactate and acidosis detected during ischemia, and the excessive generation of reactive oxygen and nitrogen species during reperfusion, can potentially activate various protein kinase pathways, including MAP kinase, JNK1/2, ERK1/2 Akt, and sodium hydrogen exchanger kinase [26]. The salvage of the myocardial cells from IR injury needs to prevent apoptosis and regulate glycogen synthesis and glucose transport via activation of the PI3k/Akt signalling axis, which mediates several functions through the phosphorylation and inactivation of GSK-3 α/β [27]. Early studies have shown that the GSK-3β inhibitor SB216763 can attenuate myocardial ischemia reperfusion injury in animals without any comorbidities with diabetes mellitus [28], or hyperlipidaemia [29]. It is well established that GSK3 modulates different cellular activities through the integration of different signalling pathways and the corresponding functional proteins [23]. In fact, more than 100 proteins have been reported to be the target of GSK3β phosphorylation, one of the underlying mechanisms involved in the preservation of mitochondrial bioenergetics functionality [30]. During the ischemic phase, the mitochondrial permeability transition pore (mPTP) is primed to open, and this process may be completed by the reperfusion phase, leading to cell death [31]. Thus, many cardio-protective strategies like pharmacological conditioning focus on suppressing the mPTP opening, and evidence suggests the critical role of GSK3β in this process [32].

In the present study, we found that PM_2.5_ exposure for a longer duration deteriorated cardiac function and impaired the heart’s ability to withstand IR stress. This negative effect of PM_2.5_ was absent with a single exposure, indicating that the associated changes were transient and reversible. Many studies have shown that long-term exposure to PM_2.5_ can impart changes in the epigenetic modifications that control different genes. For instance, a recent study demonstrated that hypermethylation of the *ADRB2* gene that encodes β2-Adrenergic receptor (β2AR) promotes the downregulation of PI3K/Akt and subsequent activation of Bcl-2/BAX and the p53 pathway [33]. Moreover, long term exposure can indeed reduce the basal expression of β2AR and VEGFR2 in heart tissues, leading to cardiac contractile dysfunction. Similarly, a few other investigators have explored the toxic effect of PM_2.5_ in zebra fish larvae and showed that PM_2.5_ can activate PI3K/akt2/mTORC1 signalling via the aryl hydrocarbon receptor or ROS-induced PTEN suppression [34]. However, in the present study, we found the opposite result, where the basal expression of PI3k/Akt/GSK3β/AMPK were downregulated by 21 days of PM_2.5_ exposure.

The negative effect of PM_2.5_ on the myocardium was prominent only when the duration of exposure was increased to 21 days, indicating the possible structural and cellular modifications mediated by SRM 2975. Accordingly, the present study result demonstrated cardiac hypertrophy only in rat hearts exposed to PM_2.5_ for 21 days. A recent study has shown that PM_2.5_ exposure can induce cardiac hypertrophy via circRNA_0001859, which suppressed miR-29b-3p, resulting in an enhanced Ctnnb1 level and activated the downstream pathway molecules like LEF1/IGF-2R [35]. Incidentally, insulin growth factor is involved in the ERK-primed inactivation of GSK-3β [36]. Additionally, the involvement of GSK-3β in the development of cardiac hypertrophy is linked with its activation, and that, in turn diminished hypertrophy in response to chronic beta-adrenergic stimulation and pressure overload [14]. In the present study, declines in the PI3K/Akt and GSK-3β levels were prominent in PM_2.5_-exposed rat hearts. In fact, PM_2.5_ can trigger the β2-adrenergic receptor (β2AR), and this persistent trigger may induce the activation of hypertrophic events which may augment the release of IL-6 [37].

Administration of GSK-3β inhibitor modulates different kinase pathways that enable the integration of signalling, and thus provides cardio-protection against IR in normal rat hearts. But persistent exposure of rats to PM_2.5_ disrupts the adaptive signalling integration that prevents the normalization of cellular events like mitochondrial function, oxidative stress, and inflammation, leading to higher IR-associated cardiac injury and prominent decline in cardiac physiology.

## 5. Conclusions

In the present study, we demonstrated that SB216763 (GSK-3β inhibitor) attenuated IR-associated cardiac injury and deteriorated cardiac physiology in rats exposed to a single dose of PM_2.5_ for 3 h. But this protection was lost when the exposure was extended to 21 days. Prolonged exposure of rats to PM_2.5_ induced the development of cardiac hypertrophy and thus reduced the responsive ability to withstand ischemia reperfusion injury. The overall cardiac dysfunction was partly associated with the elevated oxidative stress that led to mitochondrial dysfunction. Additionally, the inability of the pro-survival signalling pathways to respond optimally augmented the negative effect of prolonged PM_2.5_ exposure, and thus we need to revisit the efficiency of existing promising and established therapeutic targets used for the treatment of myocardial ischemia reperfusion injury.

## Data Availability

The datasets generated during and/or analysed during the current study are available from the corresponding author on reasonable request.

## Abbreviation

PM: Particulate matter; DPM: Diesel particulate matter; GSK3β: glycogen synthase kinase-3 beta; IR: Ischemia reperfusion injury; ROS: Reactive oxygen species; ETC: Electron transport chain; KH: Krebs-Henseleit buffer; RCR: Respiratory control ratio.
